# Calf Management: Individual or Paired Housing Affects Dairy Calf Health and Welfare

**DOI:** 10.3390/ani14111540

**Published:** 2024-05-23

**Authors:** David S. McFarland, Lorna M. McFarland, Darren J. Shaw, Alastair I. Macrae

**Affiliations:** 1 Division of Farm Animal, Veterinary Public Health and Epidemiology, Royal Dick School of Veterinary Studies and the Roslin Institute, Easter Bush Campus, Midlothian EH25 9RG, UK; darren.shaw@ed.ac.uk (D.J.S.); a.i.macrae@ed.ac.uk (A.I.M.); 2 Independent Researcher, Newtownstewart BT78 4LG, UK

**Keywords:** calf, social housing, daily live weight gain, salivary cortisol, pair, novel object, latency to feed

## Abstract

**Simple Summary:**

This study involved 138 Holstein dairy calves from one UK farm, who were either reared individually or as pairs until 2–4 weeks of age, when they were subsequently batched together. Although there were no differences in growth rate, disease treatments or mortality, the paired calves demonstrated improved behavioural indicators of calf welfare. In addition, the heaviest calf in the pair grew more quickly and displayed less fear and more exploratory behaviour than the lightest calf in the pair. Although recent advice to farmers has been to rear calves in pairs, this study suggests that this may be potentially detrimental to the smaller calf.

**Abstract:**

Previous research has indicated that preweaned dairy calves reared in pairs compared with individually have improved performance and indicators of animal welfare. One hundred and thirty Holstein female calves completed the trial, with eighty-five being allocated to paired housing and forty-five calves being allocated to individual housing. Daily live weight gain (DLWG), treatments and mortality were recorded throughout the preweaning period. Salivary cortisol, latency to feed and latency to approach a novel object were assessed at batching. There were no significant differences in DLWG, mortality and disease treatments between the average of the pair and the individually housed calves, although the pair-reared calves were quicker to approach the milk feed after batching and interacted more quickly with a novel object. The heaviest born calves within the pair had the highest DLWG from birth to weaning, with a higher percentage of calves approaching the novel object, compared with the lightest born calf within the pair. This study shows that calves within a pair may have significantly different performance and welfare during the preweaning period, with the heavier calf outperforming and displaying less fear and more exploratory behaviour than the lighter calf within a pair.

## 1. Introduction

The traditional rearing of dairy calves individually in pens or hutches allows for easier observation and management whilst preventing the transmission of infectious diseases such as diarrhoea and pneumonia. However, recent studies have shown benefits to dairy calves reared in social housing (either in groups or pairs), in comparison to housing calves individually. Improved performance has been observed in group housing systems, with improved daily live weight gain (DLWG) and solid feed intake being seen during the preweaning stage [1,2,3]. Previous work has shown that individually reared calves are more fearful in comparison to calves reared in social housing [4,5], with paired calves showing less vocalisation at weaning in comparison to individually reared calves [6,7]. These studies suggest that rearing calves in pairs or groups may equip calves to better adapt to changes in the environment or management during the preweaning period [3].

Whilst various behavioural measurements have been used to assess calf welfare, few studies have used cortisol as a measurement of the stress response in calf social housing studies [8,9,10]. As an alternative to blood sampling, salivary cortisol is a non-invasive approach, having approximately a 10% yield in cortisol in comparison to plasma [11]. Salivary cortisol samples have been shown to peak approximately sixty minutes after a stressful event in previous calf studies [11,12,13]; so, salivary cortisol is a useful tool to help assess the stress response in calves during the preweaning period.

The majority of studies to date have compared paired and individually reared calves for all of the milk rearing phase, for between six and sixteen weeks of age [2,5,7]. However, a recent study found that only 40% of UK farms made no change in housing groups during the preweaning period, with the most common change being a move from individual into group pens [14]. There is little research to date focussing on the effects of calf rearing in pairs for a limited period in early life, with early grouping during the milk rearing phase [15].

With the majority of published research using the average of the pair to assess the performance of calves during calf social housing experiments [1,16,17], no research has been published on whether both calves within the pair have the same outcome, compared with averaging the data from the pair.

The objective of this study was to assess the effect of the single and paired housing of calves on dairy calf performance and welfare during the preweaning period. The aim was to assess this by using two methods: the average of the paired calves in the pen and looking at each calf within the pair separately to assess if there was a difference between calves within the pair. With little research having been carried out on calf social housing based on group movement and batching during the milk rearing phase, as commonly carried out on UK dairy farms [14,18], this study also aimed to provide data directly relevant to UK commercial dairy farms about the pair rearing of calves. Using behavioural measurements such as latency to feed and latency to interact with novel objects would enable assessment of calf fearfulness, exploratory behaviour and welfare, especially after stressful events such as batching. Our hypothesis was that the social relationship developed from pairing calves from birth would equip them with positive attributes to help improve their welfare, with the ability to adapt and manage potentially stressful situations such as batching.

## 2. Materials and Methods

This study was carried out on a 450-cow Holstein herd based in Northern Ireland, UK. The study was approved by University of Edinburgh, Royal (Dick) School of Veterinary Studies, Veterinary Ethical Review Committee (VERC; reference number 5.21).

The required sample sizes were calculated by using Minitab 20.3 (Minitab, LLC, State College, PA, USA), using a 95% confidence interval and 0.8 power. Calculations were based on an average DLWG of 0.7 kg with a 0.2 kg difference between groups (sample size of 64 animals) and a 10% difference in morbidity based on a 30% treatment rate (sample size of 62 animals). By using an 8% average UK calf mortality rate, the required sample size of over 1000 calves meant that it was not going to be possible to have sufficient calves to assess any differences in mortality rate [19,20].

One hundred and thirty-eight female Holstein dairy calves were enrolled in the trial from 14 September 2021 to 22 January 2022. Of the 138 calves enrolled, 92 were allocated to paired housing at birth, and 46 calves were allocated to individual housing. Mortality and morbidity were the primary reason for removal of calves from the study. Calves in pairs that did not complete the full pre-batching period as a pair were both removed from the trial. The six calves removed from the study for these reasons were included in the morbidity and mortality treatment only. One calf was incorrectly sexed at birth and was thus not included in any of the data analysis, along with the other paired calf in its pen from birth. Of the one hundred and thirty-two calves that were batched in groups of twelve, ten groups contained eight paired calves and four individual calves, with one group containing seven paired calves and five individual calves. Of the one hundred and thirty Holstein female calves with full datasets that completed the trial finishing on 9 March 2022, eighty-five were pair-housed at birth and forty-five were individually housed at birth. These 130 calves were included in the analysis for DLWG, salivary cortisol, latency to feed and latency to approach a novel object. A total of 136 calves were included in the morbidity and mortality data. This included calves removed from the trial due to illness or death.

### 2.1. Calf Management

Calves were born in a group maternity pen on deep bedded straw and were removed from their dam within twelve hours of birth. Calves were fed 3.5 L of their dam’s colostrum within twelve hours of birth, with a further 3.5 L being provided within twenty-four hours of life. Two feeds of 3.5 L of the dam’s colostrum was fed on day two. Calves were then fed powdered milk replacer on day three after birth (Triple AAA Golden Maverick; Volac, Orwell, UK). This was fed at 125 g per litre, with calves being built up to 4 L fed twice daily by seven days of age (8 L in total per day). Feeding times were from 6 am to 8 am and from 4 pm to 6 pm, with milk being delivered by using the same mobile milk tank. Calves were reared on both teat feeders and buckets during the pre-batching stage, initially starting on teat feeders before being moved onto buckets in the week prior to batching. The milk feed equipment, teat feeders and milk buckets were washed on a daily basis by using warm water. Calves also had access to fresh water and starter concentrate (Thompson Calf Pride; Thompsons, Belfast, UK) from entering the pen on day one.

**Figure 1:**
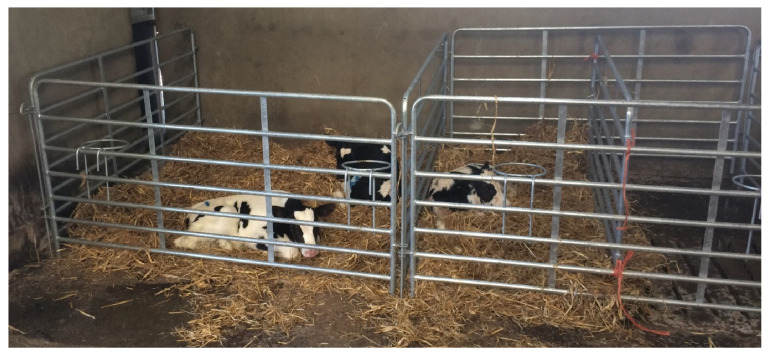
An example of a paired and an individual pen used in the study.

Holstein heifer calves were placed into either paired or individual pens based on their order of birth. For example, calf one was placed in an individual pen, calves two and three were placed in a paired pen, and calf four was placed in an individual pen (Figure 1).

**Figure 2:**
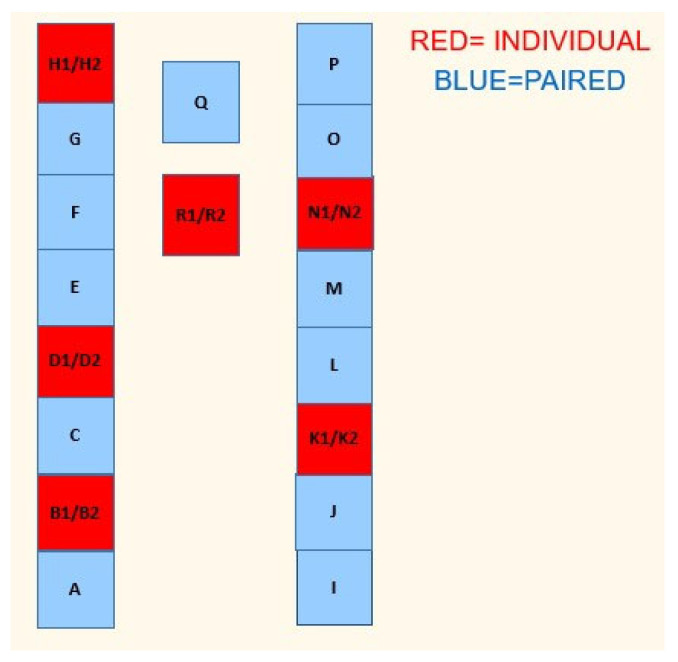
Layout of the calf shed used from birth to batching. Individual pens are in red and paired pens in blue.

All calves were housed in the same shed at birth, made up of twelve individual pens and twelve paired pens (24 paired calves in twelve pairs, 12 individually penned calves), giving 36 calves in total in the shed. The paired pens were placed throughout the shed to minimise the potential variation in the environment within the shed (Figure 2).

Pen divisions were made with metal bar panels (183 cm in length) allowing for auditory, visual and physical contact between neighbouring pens. Paired pens were 183 cm in length by 183 cm in width, and individual pens were 183 cm by 91.5 cm in width, giving both paired and individually housed calves the same floor space of 1.67 m^2^. Calves were bedded on deep barley straw, which was replenished with fresh straw daily.

**Figure 3:**
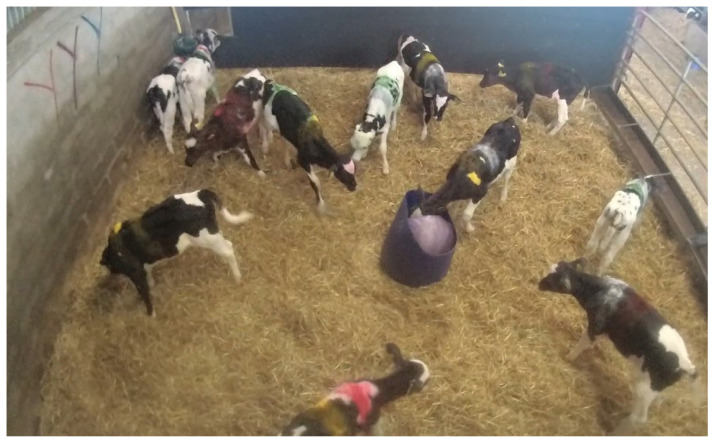
Example of calves in a batch with their unique colour identification marking during the latency to approach a novel object test.

At batching, at around 2–4 weeks of age, calves were moved into a separate batching shed in groups of twelve. Where possible, batches were made up of 4 individually housed and 8 pair-housed calves, giving 12 calves in total per pen. The batching shed consisted of eight pens 4.57 m in length and 4.27 m in width, providing 1.62 m^2^ of floor space per calf. Pen barriers between batches were made of solid boarding; therefore, nose-to-nose contact between pens was limited (Figure 3). Once calves were in their batch, there were no group changes until after weaning. Water was available in each pen via a drinker, and milk was fed from a trough with sufficient space for all twelve calves in the pen to drink at once. The trough had no dividers, with all calves in the batch having access to the total quantity of milk for the group. The milk replacer used (Triple AAA Golden Maverick; Volac, UK) was fed at one hundred and twenty-five grams per litre, with calves drinking 4 L twice daily. Calves had ad libitum access to starter concentrate and straw, which were available throughout the day and refreshed on a daily basis. Calves were bedded on deep barley straw, which was replenished with fresh straw daily. Calves were routinely disbudded post-weaning; so, no disbudding of calves occurred during the period in which the calves were on the study.

### 2.2. Body Weight and Calf Performance

Calves were weighed at three timepoints throughout the trial: at birth, the day prior to batching and one week prior to weaning. Calves were fed four litres of milk replacer twice daily whilst in the batches and completed the trial once they had their final weight recorded. Milk feeding was then reduced for one week until weaning after calves completed the study. The weighing of calves was carried out by using a Ritchie weigh crate with a fitted Tru-test digital load cell and load bars.

Calves were diagnosed with clinical disease according to standard farm protocols. These were recorded and treated accordingly, in relation to farm treatment protocols. For example, calves with respiratory disease (diagnosed primarily based on clinical signs such as laboured breathing, coughing, nasal discharge or lethargy with a rectal temperature of over 39.6 °C) were treated with 2.5 mg tulathromycin/kg bodyweight subcutaneously (Draxxin, Zoetis, Leatherhead, UK) and 0.5 mg meloxicam/kg body weight subcutaneously (Metacam 20 mg/mL solution for injection; Boehringer Ingelheim Animal Health UK Ltd., Bracknell, UK). Calves with diarrhoea (diagnosed by fluid faeces and evidence of dehydration) were treated with oral electrolytes (Life Aid Xtra; one sachet per two litres of water), 0.2 mg butylscopolamine bromide/kg and 25 mg metamizole/kg bodyweight intramuscularly (Buscopan compositum solution for injection; Boehringer Ingelheim Animal Health UK Ltd.). In addition, 8 mg procaine penicillin/kg and 10 mg dihydrostreptomycin sulphate/kg bodyweight (Pen & Strep suspension for injection; Newry, Northern Ireland) was given by intramuscular injection for three consecutive days. Mortality and reason for calves leaving the trial were also recorded.

### 2.3. Salivary Cortisol

Saliva samples were collected at three timepoints from each calf: twenty-four hours prior to the day of batching, forty-five minutes after batching and twenty-four hours after the day of batching. Calves were sampled in the same order each day, with the aim to keep the time of sampling during the day consistent between each sample, with a twenty-four-hour time-period difference between each saliva sample collected per calf. Two synthetic swabs (Salivette^®®^ Cortisol Swabs; Sarstedt, Germany) were held in the calf’s mouth by using forceps for ninety seconds. Saliva sampling took approximately forty-five minutes per group. Restraint of calves was kept to a minimum to avoid unnecessary stress, with the majority of calves willing to chew on the forceps and Salivette swabs. If calves needed further restraint, they were guided to a corner of the pen to help facilitate handling. The routine was identical for all three saliva collection timepoints. Plastic gloves and the forceps were washed and dried with disposable paper towels between each calf to avoid cross-contamination of samples. Following collection, the swabs were immediately frozen at −20 °C for subsequent analysis. Following thawing, the cotton swabs were centrifuged at 3000× *g* for 15 min to extract the saliva, and concentrations of salivary cortisol were measured by using a commercial kit (Cortisol ELISA (Saliva); ALPCO^®®^, Salem, NH, USA) by SRUC Biomarkers Laboratory, SRUC, Roslin Institute Building, Easter Bush, Midlothian, EH25 9RG, UK.

### 2.4. Behavioural Measurements

On the day prior to batching, calves were given an individual colour marking using two coloured stripes marked on each side of the thorax (Figure 3). Saliva samples were taken first on the day prior to batching, before weighing and marking with their individual colour marking. This gave each calf a unique colour marking within its batching pen, and allowed for the identification of the calf on the video recordings. The video recording of the pen was taken by using a Reveal video camera positioned to view the whole pen and subsequently recorded onto digital media (Reveal DEMS360 version 5.4 software) to allow for subsequent analysis.

Video analysis was undertaken to assess latency to feed and latency to approach a novel object. Latency to feed was assessed at both the first and second feed after batching and consisted of a five-minute video recording from the time that milk entered the trough at feeding. Calves were deemed to be drinking milk if they were in a feeding position at the trough with their mouth entering the milk, and the time during which this drinking behaviour occurred following the arrival of milk in the trough was then determined.

Latency to approach a novel object was assessed on the day of batching and consisted of a fifteen-minute video recording from the time in which a novel object was placed in the centre of the pen. This test was performed at various times throughout the day, ranging between 11 am and 10 pm. The novel object was a 55 cm diameter purple exercise ball placed within a blue open top container to help keep the novel object’s position central within the pen (Figure 3).

One person ensured that all calves within the pen were standing and then given a minute to settle, prior to placement of the novel object in the centre of the pen. Calves were deemed to have approached the novel object if they came within 30 cm of the novel object whilst showing clear interest in the novel object, and the time during which this behaviour occurred following the arrival of the novel object in the pen was then determined. During the latency to approach a novel object test, if any calves lay down, this was also recorded, and the time at which calves lay down was also recorded following the arrival of the novel object in the pen.

### 2.5. Data Analysis

All data were entered into an Excel file, with subsequent statistical analysis in Minitab 20.3 (Minitab, LLC) and R (version 3.4.4; R Foundation for Statistical Computing, Vienna, Austria). Standard errors were calculated for normally distributed data and interquartile ranges for those not normally distributed.

Four sets of mixed-effect models were established: (a) average of a pair versus single penned; (b) individuals within a pair; (c) heaviest calf at birth within a pair versus single penned; (d) lightest calf at birth within a pair versus single penned. Which batch the calves were part of was considered the random effect in all models. Linear mixed-effect models were established to assess differences in daily live weight gain and cortisol. Normality of residuals from these models was assessed prior to reporting analytical results, with cortisol levels requiring log_10_ transformation. General linear mixed-effect models with binomial errors were established to assess differences in the 4 sets of comparison for treatment of diarrhoea or pneumonia; total treatments; calves approaching their feed within 10 s or a novel object within 30 s; or calves did not approach their feed within five minutes or the novel object within 15 min. Statistical significance was taken as *p* ≤ 0.05.

## 3. Results

### 3.1. Descriptive Analyses

Of the 130 Holstein female calves that completed the trial finishing on 9 March 2022, 85 were pair-housed at birth, and 45 were individually housed at birth. Calves had a mean birthweight of 39.81 ± SEM 0.37 kg. The median time from birth to the day prior to batching was 24 (IQR: 15–31) days. The mean weight on the day prior to batching was 51.40 ± 0.62 kg. Calves were weighed prior to reducing the milk for weaning at a median age of 64 (58–69) days, with a mean weight of 80.61 ± 1.24 kg.

### 3.2. Daily Live Weight Gain

**Figure 4:**
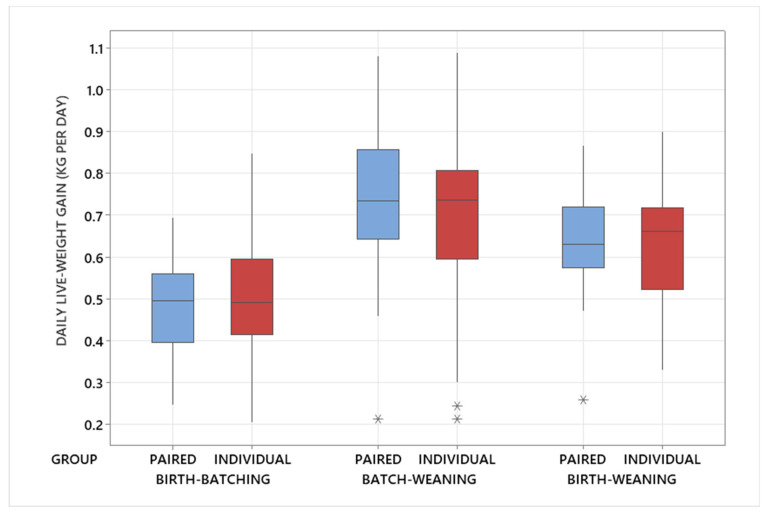
Mean daily live weight gain (DLWG) for calves either individually reared or pair-reared. The line in the box represents the median value, and the box represents the interquartile range. Outliers are identified by asterisks (*).

When using the average DLWG for the paired calves in the pen, there was no significant difference in the mean DLWG between the individually reared calves and the paired calves from birth to batching (individual versus pair: 0.50 ± 0.02 kg per day versus 0.48 ± 0.02 kg per day (mean ± SEM); *p* = 0.248; Figure 4), from batching to weaning (individual versus pair: 0.70 ± 0.04 kg per day versus 0.74 ± 0.04 kg per day; *p* = 0.390; Figure 4) or from birth to weaning (individual versus pair: 0.63 ± 0.03 kg per day versus 0.64 ± 0.03 kg per day; *p* = 0.545; Figure 4). There was no significant difference in calf birthweight between the mean of the paired calves and the individually housed calves (individual versus pair: 39.7 ± 0.58 kg versus 39.9 ± 0.60 kg; *p* = 0.731).

To assess whether there was any difference in DLWG between the paired calves, the pair within each pen were split according to their birthweight into “heavy” and “light” calves at birth. As expected, there was a significant difference in calf birthweight between the heavy and light calves within a pair (heavy versus light: 42.5 ± 0.59 kg versus 37.4 ± 0.59 kg; *p* < 0.001; Table 1). There was no significant difference in DLWG between the heavy and light calves within a pair from birth to batching (heavy versus light: 0.50 ± 0.03 kg per day versus 0.45 ± 0.03 kg per day; *p* = 0.196; Table 1) and from batching to weaning (heavy versus light: 0.77 ± 0.04 kg per day versus 0.71 ± 0.04 kg per day; *p* = 0.132; Table 1). However, there was a significant difference in DLWG from birth to weaning (heavy versus light: 0.68 ± 0.03 kg per day versus 0.61 ± 0.03 kg per day; *p* = 0.046; Table 1).

There was no significant difference in DLWG between the heavy calf in a pair at birth and the individually reared calves from birth to batching (heavy versus individual: 0.50 ± 0.03 kg per day versus 0.50 ± 0.03 kg per day; *p* = 0.986; Table 1), from batching to weaning (heavy versus individual: 0.78 ± 0.04 kg per day versus 0.70 ± 0.04 kg per day; *p* = 0.072; Table 1) or from birth to weaning (heavy versus individual: 0.68 ± 0.03 kg per day versus 0.63 ± 0.02 kg per day; *p* = 0.070; Table 1).

There was no significant difference in DLWG between the light calf in a pair at birth and the individually reared calves from birth to batching (light versus individual: 0.45 ± 0.03 kg per day versus 0.50 ± 0.03 kg per day; *p* = 0.067; Table 1), from batching to weaning (light versus individual: 0.71 ± 0.04 kg per day versus 0.70 ± 0.04 kg per day; *p* = 0.968; Table 1) or from birth to weaning (light versus individual: 0.61 ± 0.03 kg per day versus 0.63 ± 0.03 kg per day; *p* = 0.550; Table 1).

To assess whether the difference in DLWG between the paired calves was due to the difference in their birthweights, the individual penned calves were ranked according to their birthweights. The bottom 50% of the individual penned calves based on birthweight were then compared to the light calf in a pair at birth. Despite their similar median birthweights (light pair 37.4 (IQR: 35.7–39.5) kg versus bottom 50% of individual calves 37.8 (IQR: 34.9–38.9) kg; *p* = 0.880), the mean DLWG from birth to batching was significantly lower for the light calf in a pair at birth (light pair 0.45 ± 0.024 kg per day versus bottom 50% of individual calves 0.54 ± 0.031 kg per day; *p* = 0.020).

### 3.3. Mortality and Morbidity

Only 2 of the 92 paired calves and 1 of the individually housed calves died during the study. These three deaths were related to diarrhoea, with one death also showing signs of respiratory disease. There was no significant difference in treatments between the individually reared and pair-reared calves for diarrhoea (individual 70.9 ± 8.0% versus paired 71.6 ± 6.3%; *p* = 0.932), with both calves in 27 of the 43 pairs being treated for diarrhoea, and 9 pairs with neither individual treated. Diarrhoea cases were reported predominantly from birth to batching. Overall higher levels of pneumonia were reported in individual calves (23.9 ± 4.1%) versus paired (12.2 ± 8.2%), although this was not statistically significant (*p* = 0.078). Only in 1 pair did both calves have reported pneumonia, compared with 35 pairs where no pneumonia was reported. If total disease treatments were considered, there were no differences between groups (individual 81.2 ± 6.2% versus paired 77.5 ± 5.1%; *p* = 0.623), although in 29 of the 43 pairs, both calves required a treatment, compared with only 5 pairs where no treatment was applied.

No significant difference was found between the heavy and light calves within a pair for diarrhoea (heavy 65.3 ± 8.9% versus light 77.8 ± 7.4%; *p* = 0.220), pneumonia (heavy 4.8 ± 4.2 versus light 8.1 ± 6.2%; *p* = 0.465) and total disease treatments (heavy 70.4 ± 8.2 versus light 84.8 ± 6.2; *p* = 0.120).

No significant difference was found between the heavy calves in a pair at birth and the individually reared calves for diarrhoea (heavy 64.4 ± 7.6% versus individual 69.0 ± 7.1%; *p* = 0.649), pneumonia (heavy 8.0 ± 4.6% versus individual 21.9 ± 7.8%; *p* = 0.072) and total disease treatments (heavy 69.0 ± 7.1% versus individual 80.0 ± 6.0%; *p* = 0.243).

No significant difference was found between the light calves in a pair at birth and the individually reared calves for diarrhoea (light 76.2 ± 6.7% versus individual 68.9 ± 7.0%; *p* = 0.448), pneumonia (light 8.8 ± 5.7% versus individual 17.4 ± 8.9%; *p* = 0.215) and total disease treatments (light 83.3 ± 5.8% versus individual 80.0 ± 6.0%; *p* = 0.689).

### 3.4. Salivary Cortisol

**Figure 5:**
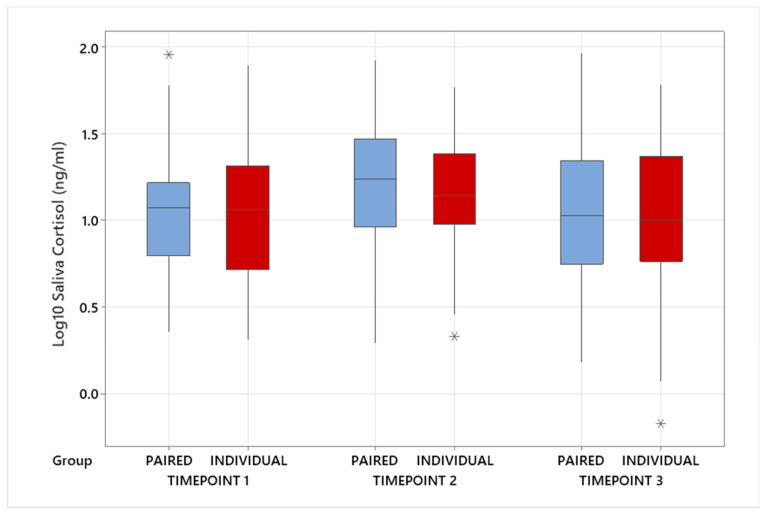
Salivary cortisol measurements in calves reared individually or in pairs. Timepoint 1: twenty-four hours prior to the day of batching. Timepoint 2: forty-five minutes after batching. Timepoint 3: twenty-four hours after the day of batching. The line in the box represents the median value, and the box represents the interquartile range. Outliers are identified by asterisks (*).

When the individually housed calves were compared to the average of the paired calves in a pen, there was no significant difference in log_10_-transformed salivary cortisol at Timepoint 1 (individual back-transformed mean = 11.4 ± 1.19 ng/mL versus paired 12.0 ± 1.19 ng/mL; *p* = 0.712; Figure 5), Timepoint 2 (individual 13.6 ± 1.16 ng/mL versus paired 18.0 ± 1.16 ng/mL; *p* = 0.070; Figure 5) and Timepoint 3 (individual 10.5 ± 1.19 ng/mL versus paired 12.3 ± 1.19 ng/mL; *p* = 0.342; Figure 5).

No significant difference was found between the heavy and light calves within a pair at birth for salivary cortisol at Timepoint 1 (heavy 12.1 ± 1.19 ng/mL versus light 12.5 ± 1.19 ng/mL; *p* = 0.857), Timepoint 2 (heavy 14. 5 ± 1.18 ng/mL versus light 21.6 ± 1.19 ng/mL; *p* = 0.085) or Timepoint 3 (heavy 11.6 ± 1.22 ng/mL versus light 13.6 ± 1.22 ng/mL; *p* = 0.505).

No significant difference was found between the heavy calves in a pair at birth and the individually reared calves for salivary cortisol at Timepoint 1 (heavy 13.5 ± 1.25 ng/mL versus individual 11.4 ± 1.22 ng/mL; *p* = 0.351), Timepoint 2 (heavy 15.0 ± 1.21 ng/mL versus individual 13.6 ± 1.16 ng/mL; *p* = 0.642) and Timepoint 3 (heavy 11.0 ± 1.27 ng/mL versus individual 10.5 ± 1.22 ng/mL; *p* = 0.807).

No significant difference was found between the light calves in a pair at birth and the individually reared calves for salivary cortisol at Timepoint 1 (light 11.5 ± 1.25 ng/mL versus individual 11.4 ± 1.21 ng/mL; *p* = 0.988) and Timepoint 3 (light 13.9 ± 1.26 ng/mL versus individual 10.5 ± 1.19 ng/mL; *p* = 0.237), but there were differences at Timepoint 2 (light 21.3 ± 1.20 ng/mL versus individual 13.7 ± 1.15 ng/mL; *p* = 0.015).

### 3.5. Latency to Feed

There were 6 individual calves and 14 paired (8 heavy and 6 light) calves that did not approach the first feed after batching during the observation period. Of the 39 individual calves that did approach the first feed after batching, they did so in a median time of 15.0 s [IQR: 0.0–73.0], whereas the 71 paired calves took a median time of 26.0 s [0.0–149.5].

There was one individual calf and six paired (four heavy and two light) calves that did not approach the second feed after batching during the observation period. Of the 44 individual calves that did approach the second feed after batching, they did so in a median time of 6.0 s [IQR: 0.0–21.0]. The 79 paired calves took a median time of 6.0 s [0.0–16.0].

A significantly higher proportion of the paired calves (either one or both of the calves in the pair) approached the milk within 10 s of feeding at both the first milk feed after batching (individual calves versus paired; 44.4% versus 69.5%) and the second milk feed after batching (60.0% versus 90.5%) (Table 2). However, there were no significant differences in the proportion of calves that did not approach the milk feed within 5 min, or between the heavy and light calves in the pair.

### 3.6. Latency to Approach a Novel Object

There were 6 individual calves and 14 paired (3 heavy and 11 light) calves that did not approach the novel object during the observation period. Of the 39 individual calves that did approach the novel object, they did so in a median time of 69.0 s [IQR: 23.0–255.0] whereas the 71 paired calves took a median time of 90 s [35.0–470.0].

A significantly higher proportion of the paired calves (either one or both of the calves in the pair) approached the novel object within 30 s (individual calves versus paired: 26.5% versus 64.2%) (Table 3). Whilst there were no differences in the speed at which the heavy and light calves approached the novel object, more of the lighter calves did not approach the novel object within 15 min compared with the heavier calves in the pair (heavier calves 7.3% versus lighter calves 26.8%) (Table 3).

## 4. Discussion

To our knowledge, this is the first study to investigate the effects of paired housing by both using the average of the pair and looking at each calf within a pair separately. The majority of previous studies on the effects of pair-reared calves have used the average of the pair in their analysis [21,22,23]. When using the average measurement for the two calves in the pair, this study showed no significant differences between individual and pair-reared calves for the first 2–4 weeks of life in terms of average DLWG, morbidity, mortality and salivary cortisol measurements. However, pair-reared calves were quicker to approach the milk feed after batching and to interact with the novel object. When assessing the individual calves within the pair, there were significant differences indicating that the higher-birthweight calf consistently outperformed and displayed less fear and more exploratory behaviour than the lower-birthweight calf within the pair.

In this study, no significant difference was found between the average of the paired and individually housed calves for DLWG, similar to previous work [6,24]. However, a significant difference was found in DLWG when the paired calves were assessed separately. DLWG during the pre-weaning period can have long-term effects on age at first calving, milk production, fertility and longevity within the herd [25,26,27], highlighting the importance of understanding the social relationship occurring between calves in a pair.

Little previous research has used salivary cortisol as an indicator of stress in calves during the pre-weaning period. One study [28] assessed salivary cortisol around weaning, with some studies using salivary cortisol when assessing stress associated with castration, dystocia and heat stress in calves [29,30,31]. Salivary cortisol has been used to show that regrouping can be a stressful event for six-month-old dairy heifers [32]. This is similar to our study, where batching was found to have a significant relationship with salivary cortisol. Little research has been carried out to date using salivary cortisol to assess stress at batching at 2–4 weeks of age, as was carried out in this study. We found a significant difference in salivary cortisol between the light and individual calves at Timepoint 2. Given that salivary cortisol is mainly affected by severe acute stress [33], it may be difficult to determine if the significant differences found were due to stress or related to the exercise or excitability of the calves being grouped in a new environment with unfamiliar calves. Research in stallions and geldings has found that cortisol levels increase with exercise, and in stallions, cortisol levels also increase with excitement due to the presence of a mare [34], indicating that there may be other factors that influence salivary cortisol levels in calves. This is a limitation of measuring cortisol, as it can be affected by other factors, such as exercise. However, given that the lighter calf in the pair at birth also showed more fear and less exploratory behaviour in the latency to approach a novel object, the salivary cortisol results represent further evidence suggesting a significant difference in the journey of two calves within a pair from birth to weaning.

Latency to feed can be used to assess how well calves adapt to new situations, such as batching, learning where the feed is in a new environment and showing the social capability to approach the feed in a group setting [35,36]. One study [37] found that visual contact alone is not sufficient for calves to develop social bonds and that physical contact among calves is necessary. Physical contact through the pen boundaries with neighbouring calves in our study meant that even individually housed calves had a degree of physical contact with another calf. Our study found a significant difference in latency to feed between paired and individually housed calves for the first and second feed after batching. This is different from other studies [35,38], where no difference was found in latency to feed in paired and individually housed calves. This may be explained by all calves on our study having auditory, visual and physical contact with other calves; however, calves within a pair had significantly more physical contact within their pen with another calf. One study [5] found that paired or individually housed calves with physical contact through the pen boundary with other calves had a lower heart rate than individual calves with no physical contact with other calves during a novel-environment test. This shows that physical contact through the pen boundaries may allow individually housed calves to develop some social skills similar to paired calves, but not to the same level. Competition for feed within the pair may also help explain why more of the paired reared calves approached the milk feed quicker after batching.

Latency to approach a novel object can be a useful indicator of fear and exploratory behaviour in calves [39]. In our study, a significant difference was found between the heavy and light calves within a pair for latency to approach a novel object, with more of the lighter calves not interacting with the novel object. A significant difference was also found with paired calves approaching the novel object more quickly in comparison to individually housed calves. Few studies have assessed calves approaching a novel object within a group [40], as carried out in this study. Social housing has been found to affect play and exploratory behaviour, promoting positive calf welfare [41]. One study [42] found that the age of the calf can change their behaviour with a novel object, potentially becoming more responsive to new situations as they get older during the pre-weaning period.

Some studies have shown the benefits that paired housing can have on improving a calf’s competitiveness to feed at three weeks of age [43] and after weaning [38], which is in agreement with our results. No studies to our knowledge have assessed if calves being pair-housed from birth develop a relationship that allows one of the paired calves to benefit in performance and welfare to the detriment of the other paired calf, potentially related to competitiveness for feed within the pen. Some of the literature [44] has found pairing calves from birth to affect their personality, with calves becoming bolder than individually housed calves, while personality has been linked with feeding behaviours in calves around weaning [45]. Some studies have shown personality traits among calves to be different [46]. One study showed that some calves can have a more active and exploratory personality than others [47]. This could play a role in how calves interact and adapt to new situations such as batching, with personality traits being suggested to be considered when forming social pairings or groups [48]. In this study, calves were fed through a teat before changing onto buckets, to allow for a smoother transition to the milk troughs used when group-housed. Although cross-suckling was not reported to be an issue in this study, competition at the teats was reported within the paired pens in the first 2–4 weeks of life prior to batching, particularly once one calf had finished the milk in its teat feeder. Milk ingestion stimulates the calf’s sucking motivation and declines 10–15 min after a milk meal [49], with the competition seen at the teat feeders likely linked to calves wanting to satisfy their motivation to suck. Some studies have shown that competition is increased with teat feeders in comparison to buckets [50] and that calves are more likely to suck teats when milk is involved [51], potentially increasing competition at the teat feeders in paired housing. Ad libitum milk feeding has also been shown not to remove the motivation for non-nutritive sucking in bucket-fed calves [52] but high milk allowances do help reduce cross-suckling [51].

Potentially, feeding calves on a high milk allowance through a bucket early in life may help reduce competition in pair-housed calves while allowing access to a teat for each calf to help satisfy non-nutritive sucking in the 10–15 min period after milk feeding. This may help provide a more uniform experience for both pair-housed calves within a pen to obtain the benefits social housing can offer, such as improved solid feed intake and calves becoming less fearful [3,5]. Competition for feed may be affecting the lighter calf at birth in the pair, as it is more likely to be dominated by the heavier calf. This negative experience for the lighter calf could, therefore, be linked with their lower DLWG, increased fear and less exploratory behaviour shown through the novel object approach test.

Competition at feeding, feeding method, delaying age at pairing [53] and calf personality are areas for future research in paired housing and may explain some of the differences seen in DLWG, feeding behaviour and approaching a novel object when calves within a pair were looked at separately in this study.

This study was carried out on a working dairy farm utilising commonly used calf management practices within the UK. One of the limitations within this study is that only one farm with a set calf management protocol was used in the trial, to minimize management variation. Further research work would be beneficial on farms with different calf management practices, such as spring calving, automatic calf feeders or use of milk bars with dividers allowing for an allocated amount of milk per calf, to assess if similar results are found.

## 5. Conclusions

This study showed the benefits of paired housing in comparison to individual housing, with less fear, more exploratory behaviour and a better ability to adapt to a new environment being found during the behavioural tests. This is combined with no negative effects on DLWG, morbidity and mortality. The area of concern found in this study is within a pair, where one calf will thrive in both performance and welfare to the detriment of the other calf when paired from birth. This was shown by a significant difference in DLWG and approaching a novel object between the calves within a pair. A significant difference was also found in salivary cortisol between the light and individual calves. More research is required to determine if paired housing can be used for allowing calves within a pair to have more uniform growth and positive welfare experience. This study shows the risk of using the average of the pen when assessing calf performance and welfare in paired housing, with looking at the paired calves separately being recommended.

## Figures and Tables

**Table 1:** DLWG and weight for different stages during pre-weaning between individual and pair-reared calves.

	Birthweight (kg) Mean ± SEM	Birth to Batching DLWG (kg per day) Mean ± SEM	Batching Weight (kg) Mean ± SEM	Batching to Weaning DLWG (kg per day) Mean ± SEM	Weaning Weight (kg) Mean ± SEM	Birth to Weaning DLWG (kg per day) Mean ± SEM	Time in Days from Birth to Weaning Median (IQR)
Individual (I) vs. Paired Average (PA)	I = 39.7 ± 0.58	I = 0.50 ± 0.02	I = 51.95 ± 0.97	I = 0.70 ± 0.04	I = 80.09 ± 2.07	I = 0.63 ± 0.03	I = 65 (58–70)
	PA = 39.9 ± 0.60	PA = 0.48 ± 0.02	PA = 51.18 ± 0.89	PA = 0.74 ± 0.04	PA = 80.89 ± 1.88	PA = 0.64 ± 0.03	PA = 64 (59–69)
	*p* = 0.731	*p* = 0.248		*p* = 0.390		*p* = 0.545	
Heavy (H) vs. light (L)	H = 42.5 ± 0.59	H = 0.50 ± 0.03	H = 54.66 ± 1.09	H = 0.77 ± 0.04	H = 85.94 ± 2.03	H = 0.68 ± 0.03	H = 64 (59–69)
	L = 37.4 ± 0.59	L = 0.45 ± 0.03	L = 47.79 ± 0.97	L = 0.71 ± 0.04	L = 76.30 ± 2.17	L = 0.61 ± 0.03	L = 64 (59–69)
	*p* < 0.001	*p* = 0.196		*p* = 0.132		*p* = 0.046	
Heavy (H) vs. individual (I)	H = 42.5 ± 0.59	H = 0.50 ± 0.03	H = 54.66 ± 1.09	H = 0.78 ± 0.04	H = 85.94 ± 2.03	H = 0.68 ± 0.03	H = 64 (59–69)
	I = 39.7 ± 0.58	I = 0.50 ± 0.03	I = 51.95 ± 0.97	I = 0.70 ± 0.04	I = 80.09 ± 2.07	I = 0.63 ± 0.02	I = 65 (58–70)
	*p* = 0.000	*p* = 0.986		*p* = 0.072		*p* = 0.070	
Light (L) vs. individual (I)	L = 37.4 ± 0.59	L = 0.45 ± 0.03	L = 47.79 ± 0.97	L = 0.71 ± 0.04	L = 76.30 ± 2.17	L = 0.61 ± 0.03	L = 64 (59–69)
	I = 39.7 ± 0.58	I = 0.50 ± 0.03	I = 51.95 ± 0.97	I = 0.70 ± 0.04	I = 80.09 ± 2.07	I = 0.63 ± 0.03	I = 65 (58–70)
	*p* = 0.007	*p* = 0.067		*p* = 0.968		*p* = 0.550	

**Table 2:** Latency to feed from milk trough following batching between individual and pair-reared calves.

	First Milk Feed after Batching		Second Milk Feed after Batching	
	Percentage of Calves Approaching Milk within 10 s	Percentage of Calves That Did Not Approach within 5 min	Percentage of Calves Approaching Milk within 10 s	Percentage of Calves That Did Not Approach within 5 min
Individual (I) vs. Paired Average (PA)	I = 44.4 ± 11.4%	I = 13.3 ± 5.1%	I = 60.0 ± 7.3%	I = 2.2 ± 2.2%
	PA = 69.5 ± 10.4%	PA = 9.5 ± 4.5%	PA = 90.5 ± 4.5%	PA = 2.4 ± 2.4%
	*p* = 0.032	*p* = 0.579	*p* = 0.002	*p* = 0.961
Heavy (H) vs. light (L)	H = 51.1 ± 8.3%	H = 15.1 ± 7.8%	H = 65.9 ± 7.4%	H = 9.8 ± 4.6%
	L = 43.6 ± 8.3%	L = 10.1 ± 7.8%	L = 78.0 ± 6.5%	L = 4.9 ± 3.4%
	*p* = 0.502	*p* = 0.533	*p* = 0.222	*p* = 0.405
Heavy (H) vs. individual (I)	H = 51.0 ± 9.2%	H = 18.6 ± 6.8%	H = 65.9 ± 7.4%	H = 9.8 ± 4.6%
	I = 44.4 ± 8.8%	I = 13.3 ± 5.4%	I = 60.0 ± 7.3%	I = 2.2 ± 2.2%
	*p* = 0.556	*p* = 0.441	*p* = 0.575	*p* = 0.171
Light (L) vs. individual (I)	L = 43.3 ± 8.9%	L = 14.6 ± 5.5%	L = 78.0 ± 6.5%	L = 4.9 ± 3.4%
	I = 44.4 ± 8.5%	I = 13.3 ± 5.1%	I = 60.0 ± 7.3%	I = 2.2 ± 2.2%
	*p* = 0.922	*p* = 0.862	*p* = 0.075	*p* = 0.513

**Table 3:** Latency to approach a novel object between individual and pair-reared calves.

	Percentage of Calves Approaching Object within 30 s	Percentage of Calves That Did Not Approach within 15 min
Individual (I) vs. Paired Average (PA)	I = 26.5 ± 9.6%	I = 13.3 ± 5.1%
	PA = 64.2 ± 11.0%	PA = 4.2 ± 3.3%
	*p* = 0.003	*p* = 0.182
Heavy (H) vs. light (L)	H = 46.3 ± 8.1%	H = 7.3 ± 4.1%
	L = 26.5 ± 7.2%	L = 26.8 ± 6.9%
	*p* = 0.069	*p* = 0.027
Heavy (H) vs. individual (I)	H = 44.6 ± 12.1%	H = 7.3 ± 4.1%
	I = 26.5 ± 9.9%	I = 13.3 ± 5.1%
	*p* = 0.112	*p* = 0.369
Light (L) vs. individual (I)	L = 26.5 ± 7.8%	L = 26.8 ± 6.9%
	I = 26.5 ± 7.9%	I = 13.3 ± 5.1%
	*p* = 0.646	*p* = 0.123

## Data Availability

The raw data supporting the conclusions of this article will be made available by the authors upon request.
